# The role of neutrophils in cancer

**DOI:** 10.1093/bmb/ldy029

**Published:** 2018-08-22

**Authors:** Robert Grecian, Moira K B Whyte, Sarah R Walmsley

**Affiliations:** Medical Research Council Centre for Inflammation Research, The Queen's Medical Research Institute, The University of Edinburgh, 47 Little France Crescent, Edinburgh, UK

**Keywords:** neutrophil role, cancer, malignancy, tumourigenesis, tumour microenvironment, tumour-associated neutrophil, low-density neutrophil, myeloid-derived suppressor cells

## Abstract

**Introduction:**

It has been known for some time that neutrophils are present in the tumour microenvironment, but only recently have their roles been explored.

**Sources of data:**

Comprehensive literature search of neutrophils and cancer (PubMed, Google Scholar and CrossRef) for key articles (systematic reviews, meta-analyses, primary research). References from these articles cross-checked for additional relevant studies.

**Areas of agreement:**

Neutrophils are a heterogeneous population with both pro- and antitumour roles, and display plasticity. Several neutrophil subpopulations have been identified, defined by a combination of features (density, maturity, surface markers, morphology and anatomical site).

**Areas of controversy:**

Limitations in translating murine tumour models to human pathology and paucity of human data. Consensus in defining human neutrophil subpopulations.

**Growing points:**

Neutrophils as therapeutic targets and as possible playmakers in the biological response to newer targeted cancer drugs.

**Areas timely for developing research:**

Understanding the metabolic programming of neutrophils in the tumour microenvironment.

## Introduction

The ability of malignant cells to establish themselves in a niche and subsequently metastasize is not entirely dependent on their own intrinsic cellular signalling pathways; complex interactions with a myriad of immune cells in the tumour microenvironment are a key.^1^ It has taken time for neutrophils to be recognized as an active player (rather than spectator) in the immune response to malignancy.^2^ This review will highlight the progress made to date in understanding their role in cancer, emphasizing areas where more work is needed.

## The association between neutrophils and cancer prognosis

### Neutrophil-to-lymphocyte ratio

Peripheral blood neutrophil counts are increased in patients with cancer. Tumours produce granulocyte colony-stimulating factor (G-CSF) which skews the neutrophil retention/release balance in bone marrow, leading to this increase in blood neutrophils.^3^ G-CSF downregulates chemokine receptor type 4 (CXCR4) expression in human myeloid lineage cells, reducing their response to the bone marrow retention signal stromal cell-derived factor 1 (SDF-1).^4^

Many research groups have investigated whether the number of neutrophils present in peripheral blood correlates with patient outcome. Most have done this using neutrophil-to-lymphocyte ratio (NLR). A meta-analysis of 100 such studies by Templeton *et al.* comprising over 40 000 patients, showed NLR > 4 to be associated with worse overall survival, cancer-specific survival, progression-free survival and disease-free survival.^5^ This was seen in all types and stage of cancer.

### Intra-tumoural neutrophils

The association between peripheral blood neutrophils and survival does not however give any information about what might be happening at the tumour site itself i.e. whether tumour-associated neutrophils (TANs) are associated with outcome. A meta-analysis of nearly 4000 patients has shown high levels of intra-tumoural neutrophils to be associated with unfavourable survival.^6^ In addition, Gentles *et al.* used a computational approach to analyse bulk tumour transcriptomes in order to infer the frequency of different immune cell populations (including neutrophils) in over 3000 solid tumours (14 cancer types). They found intra-tumoural neutrophils to be the most adverse prognostic cell population.^7^

### Neutropaenia

In direct contrast, neutropaenia in patients undergoing chemotherapy has been shown (by meta-analysis) to be beneficial to survival.^8^ This may of course just be a reflection of adequate toxicity of the drug being achieved to kill tumour cells, however the question does arise as to whether the direct effect on neutrophils themselves is beneficial. This is clinically relevant, as the use G-CSF in patients who are profoundly neutropaenic post-chemotherapy (used with the aim to prevent sepsis) could in fact be detrimental to longer term clinical outcome. Indeed, it has been shown that G-CSF may promote metastatic disease via neutrophils forming a pre-metastatic niche.^9^

### What about neutrophil function, site and regulation?

Measuring the number of neutrophils in the blood and/or in the tumour of cancer patients and associating this with survival is of course quite a crude measure and does not give any indication regarding mechanism. It must also be remembered that blood neutrophil levels increase under other conditions, such as infection. Within the same patient, neutrophils may display varying roles at different sites. Furthermore, appropriate inflammatory responses are dependent upon a functioning balance of neutrophil production, release from bone marrow, recruitment to the site of injury and clearance. Dysregulation of this homeostatic process, for example, by tumour-derived G-CSF, could perpetuate malignancy.

## Neutrophil polarization at the tumour site

### Transforming growth factor beta and interferon beta have opposite effects on TAN polarization in mice

Fridlender was the first to suggest that TAN may be polarized to N1 (antitumour) or N2 (pro-tumour) phenotypes, in a similar manner to macrophages. Using tumour-bearing mice, he demonstrated that transforming growth factor beta (TGF-β) blockade favoured the accumulation of N1 TAN that were morphologically and functionally different to N2 TAN. N1 TAN had hypersegmented nuclei in contrast to the nuclei of N2 TAN that were circular. N1 TANs were cytotoxic to tumour cells via an oxygen radical-dependent mechanism and had increased tumour necrosis factor alpha (TNF-α) and intercellular adhesion molecule 1 (ICAM-1) expression, whereas N2 TAN expressed high levels of arginase which is known to suppress T cell immunity. Of key importance, TGF-β blockade promoted a T cell antitumour response.^10^ Using IFN-β_1_^−/−^ tumour-bearing mice, the Jablonska group have shown interferon beta (IFN-β) to have the opposite effect to TGF-β on TAN polarization, i.e. IFN-β promotes antitumour N1 TAN.^11^

### Limitations of N1 vs N2 model

It should be noted that the work described above in murine models is yet to be replicated in human TAN. Furthermore, in a similar manner to M1 vs M2 macrophage classification in malignancy, it may be that a binary N1/N2 classification of neutrophils is an oversimplification.^12^ It seems increasingly likely that N1 and N2 represent laboratory extremes of a biological continuum, with plasticity dependent upon the local environment. Therefore rather than focusing on N1/N2, we should possibly instead be defining neutrophils by the distinct functional phenotypes/subpopulations that aid or abate the process of tumourigenesis (e.g. proliferation, angiogenesis, invasion, immunosuppression and metastatic seeding) in the different microenvironments (i.e. primary tumour, circulation, pre-metastatic and metastatic).

## Neutrophil roles in aiding or abating tumourigenesis

### Tumour proliferation

Neutrophil elastase (NE) has been shown to promote tumour proliferation. Houghton *et al.* showed that NE is taken up by tumour cells, where it degrades insulin receptor substrate-1 (IRS-1). Lower levels of IRS-1 were associated with an increase in the interaction between phosphatidylinositol 3-kinase (PI3K) and the potent mitogen platelet-derived growth factor receptor (PDGFR), which directed the PI3K axis to favour tumour proliferation.^13^

In contrast, neutrophils can also induce lysis of tumour cells via hypochlorus acid produced from reactive oxygen species (ROS).^14^ Of note, the MET proto-oncogene is expressed in neutrophils and is required for neutrophil chemoattraction and cytotoxicity towards tumour cells in response to its ligand hepatocyte growth factor.^15^ Neutrophils can kill tumour cells via TNF-α expression.^11^ Furthermore, neutrophils stimulated by interferons release tumour necrosis factor-related apoptosis-inducing ligand (TRAIL) which induces tumour cell apoptosis.^16^

### Angiogenesis and invasion

The angiogenic and invasive mechanisms of matrix metallopeptidase 9 (MMP-9), vascular endothelial growth factor (VEGF) and Bv8 (prokineticin) have been previously described.^17,18^ It is thought that neutrophils may drive angiogenesis in malignancy by providing a significant source of MMP-9 which acts to release VEGF from the extracellular matrix (ECM).^19,20^ In addition to roles in angiogenesis, MMP-9 is also postulated to aid the direct invasion of tumour cells via degradation of ECM/basement membrane.

Of note, it has been shown that neutrophil extracellular traps (NETs), formed during neutrophil death, and composing of chromatin, NE and myeloperoxidase, have a role in angiogenesis, by stimulating vascular endothelial cells to release proangiogenic cytokines.^21^

Countering the above mechanisms, neutrophils can also have an opposing function with regard to angiogenesis; it has been reported that neutrophils can be conditioned *ex vivo* to release the antiangiogenic isoform of VEGF (VEGF-A_165b_),^22^ but it is yet to be proven if this occurs *in vivo*.

### Immunomodulation

As previously mentioned, neutrophils are known to express arginase. Arginase degrades arginine, an essential amino acid important in many cellular processes, e.g. the proliferation of T cells. High arginase levels can be found in the tumour microenvironment and result in inhibition of T cell receptor expression and antigen-specific responses, aiding tumour evasion.^23^ Neutrophils have been widely reported to suppress T cell proliferation in *ex vivo* studies, with Coffelt *et al.* demonstrating this is inducible nitric oxide synthase (iNOS)-dependent,^24^ but it must be noted that recently concerns have arisen about the accuracy of these assays when T cell activating microbeads have been used.^25^ Neutrophils have also been shown to induce the apoptosis of CD8 T cells in a TNF-α- and nitric oxide (NO)-dependent manner.^26^ In addition, neutrophils can suppress T cells via programmed death ligand 1 (PD-L1).^27^ Moreover, neutrophil depletion studies suggest that they may act to reduce the effectiveness of PD1 immunotherapy.^28^ Finally, neutrophils recruit regulatory T cells into tumours via secretion of chemokine ligand 17 (CCL17), which may inhibit antitumour immunity.^29^

Whilst there appears to be an ever-expanding list of ways in which neutrophils immunosuppress in the tumour microenvironment, nonetheless neutrophils can also be immunostimulatory and antitumour. Neutrophils can have a role in antigen presentation,^30^ can stimulate T cell proliferation^31^ and suppress pro-tumoural IL-17 γδ T cells via ROS.^32^

### Extravasation and metastatic seeding

NETs have been shown to sequester circulating tumour cells at distant sites and promote metastasis.^33^ Extravasation into tissues is aided by interactions between β_2_ integrin on neutrophils and ICAM-1 on tumour cells, promoting anchoring to the vascular endothelium.^34^ Tumour-derived G-CSF can initiate a pre-metastatic environment in distant organs, mobilizing neutrophils from the bone marrow to swarm at the metastatic site before tumour cells arrive.^35^

Conversely, Granot *et al.* showed that tumour-entrained neutrophils (i.e. stimulated by the primary tumour) can inhibit metastatic seeding via hydrogen peroxide (H_2_O_2_) killing of disseminated tumour cells.^36^ It has recently been shown that H_2_O_2_ from neutrophils kills tumour cells by triggering a lethal influx of calcium via transient receptor potential cation channel subfamily M member 2 (TRPM2), an H_2_O_2_-dependent calcium-permeable channel expressed on cancer cells.^37^

## Defining neutrophil subpopulations by phenotypic features: density, maturity, surface markers, morphology and anatomical site

**Fig. 1:**
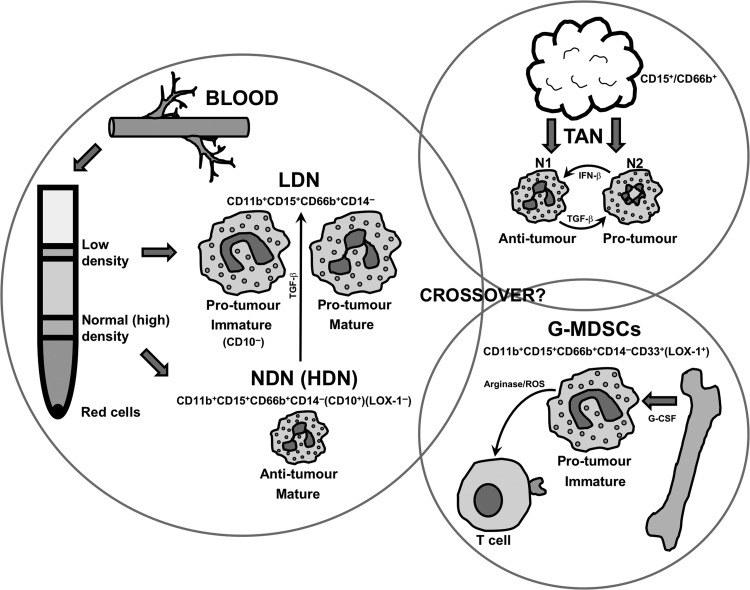
Neutrophil subpopulations defined by density, maturity, surface markers, morphology and anatomical site. At present, it is unclear whether there is any crossover between LDN, N2 TAN and G-MDSC populations. LDN, low-density neutrophils; NDN, normal (high)-density neutrophils; TAN, tumour-associated neutrophils; G-MDSCs, granulocytic myeloid-derived suppressor cells.

It is clear from earlier sections in this review that neutrophils display heterogeneity and plasticity of function in malignancy. But what other features define these neutrophils that are pro- or antitumour? Eruslanov and Fridlender groups have carried out seminal work in the last few years where functional neutrophil subpopulations have been distinguished further by density, maturity, surface markers, morphology and anatomical site (Fig. 1).^30,31,38–40^

### Peripheral blood neutrophils

*Ex vivo*, neutrophils are commonly isolated from the blood by discontinuous density gradient. This results in the formation of a red cell pellet, a normal-density (sometimes referred to as ‘high density’ in the literature) layer of cells and a low-density layer of cells. Neutrophils are usually the predominant cell type in the normal-density layer (normal-density neutrophils, NDNs) and peripheral blood mononuclear cells (PBMCs) are found in the low-density layer.^41^ In malignancy, there is an expanded population of neutrophils in the low-density layer (low-density neutrophils, LDNs), which can be further defined by maturity/morphology. LDNs are large and are either immature with a banded/ring nuclei, or mature with segmented nuclei. All LDNs are pro-tumour, displaying immunosuppressive properties. NDNs are small and mature with segmented nuclei and are antitumour. Of note, neutrophils from patients with malignancy resist apoptosis compared with healthy donor neutrophils. Finally, murine models showed LDN to originate from the bone morrow, but of interest TGF-β could also mediate the transition of HDN to LDN (displaying plasticity).^39,40^

### Tumour-associated neutrophil

The features of TAN in murine models have already been described. In humans, TANs in early-stage cancer are mature with segmented nuclei and are antitumour, activating T cell responses. Study of TAN from more advanced malignancies is proving more difficult, due to lack of availability of tissue to study (patients with advanced cancer do not typically undergo surgical resection).^30,31,38^

### Neutrophil surface marker immunotyping (human)

To date, it has proven difficult to distinguish between the neutrophil subpopulations described above, by surface marker immunotyping. Furthermore, there have been difficulties when attempting to compare neutrophil subpopulations found in murine models with those found in humans.^42^ However, most groups use CD11b^+^CD15^+^CD66b^+^CD14^−^ to identify neutrophils in humans. In addition, CD10 is proving to be a key marker for the maturation and suppressive potential of neutrophils. Indeed, going forward CD10 may reduce the need to use density gradients to define neutrophil populations.^43^

### Myeloid-derived suppressor cells

The name myeloid-derived suppressor cells (MDSCs) was originally coined over 10 years ago to describe a group of myeloid cells with immunoregulatory activity, i.e. suppressed antitumour T cell functions (via arginase and ROS). Broadly speaking, they compose two phenotypical/morphological groups of cells: those similar to neutrophils (granulocytic/polymorphonuclear MDSCs or G-MDSCs/PMN-MDSCs) and those similar to monocytes (M-MDSCs). It is thought that during chronic inflammatory processes, such as malignancy, there is a persistent signal to recruit neutrophils and monocytes from the bone marrow (e.g. GM-CSF, G-CSF, M-CSF). As time goes on, the rate of demand on the bone marrow is such that these recruited cells are increasingly immature and have aberrant function. These cells are MDSC, and as cancers progress they form a greater proportion of circulating cells. In humans as G-MDSCs and neutrophils can both be defined by the surface markers CD11b^+^CD14^-^CD15^+^(or CD66b^+^)CD33^+^, it has been difficult to distinguish between them. However, it is known that G-MDSCs are found in the low-density fraction of peripheral blood. Furthermore, G-MDSCs express lectin-type oxidized LDL receptor 1 (LOX-1) and so this marker may be used to distinguish them from neutrophils, without the need for a density gradient.^44,45^

There is debate as to how closely neutrophils and G-MDSCs are related.^46^ It seems increasingly likely that in humans, immature peripheral blood LDN and G-MDSCs are one and the same. However, mature peripheral blood LDN appear to be different to G-MDSCs. Furthermore, within tumours themselves the relationship between G-MDSCs and TAN is entirely unknown.^47^

## Discussion

### Areas of agreement

Neutrophils are no longer seen as a simple first responder cell; they have complex multifaceted roles in all stages of malignancy with both pro- and antitumour roles. In cancer, neutrophils are a heterogeneous population and display plasticity. Several neutrophil subpopulations have been identified and are currently defined by a combination of features; density, maturity, surface markers, morphology and anatomical site.

### Summary of what is known (Fig. 2)

**Fig. 2:**
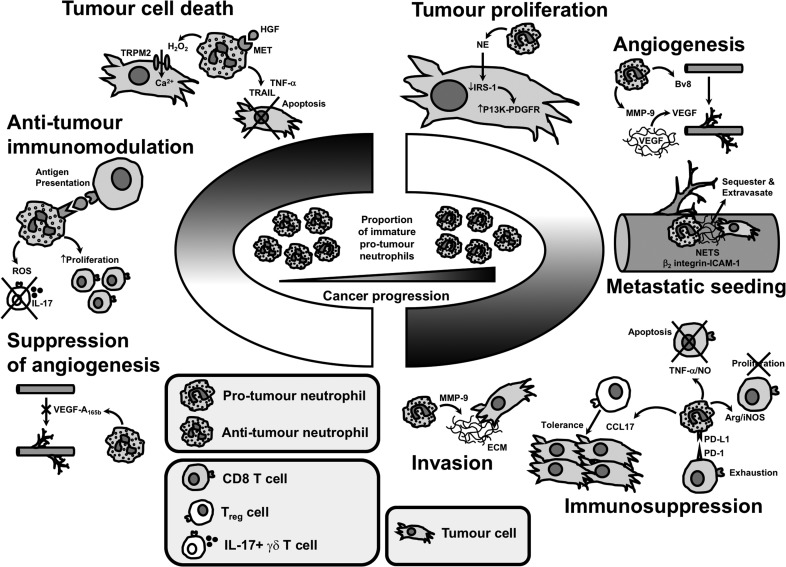
Mechanisms through which neutrophil subpopulations aid or abate tumourigenesis. The proportion of pro- and antitumour neutrophils at any one time is influx, with a degree of plasticity and a spectrum of activity. However, for cancers to progress this balance must begin to favour immature pro-tumour neutrophils.

Our summary figure (Fig. 2) aims to define neutrophils by the distinct functional subpopulations that aid or abate the process of tumourigenesis, reflecting a biological continuum/spectrum rather than focusing on N1/N2 laboratory extremes. Akin to the ‘macrophage wheel’ used by Qian and Pollard*.*^12^

### Areas of controversy

A recent review article by the Eruslanov group highlights the ongoing areas of controversy regarding the role of neutrophils in cancer.^42^ These mainly relate to (i) the limitations of translating murine tumour models to human pathology and (ii) the lack/quality of human data.

The most common form of murine model is transplantable tumours, i.e. immortal cell lines that have undergone years of selective pressure and are chosen based on their ability to grow quickly when injected into the mouse. These tumours by definition have a very different natural history to the gradual evolution of a natural tumour and therefore really only represent the later stages of malignancy. There are no shared neutrophil cellular markers between mouse and man. We do not know if immature LDN, G-MDSCs and N2 are the same cell population or not. N1/N2 polarization has not been shown in human TAN, and it seems more likely that in fact there will be a continuum of behaviour rather than these two extremes. Are N2 neutrophils just immature cells recruited as a consequence of cancer and not a contributing cause?

There is a lack of human tissue available from the later stages of cancer, as patients do not routinely have surgery. Furthermore, whilst peripheral blood may be more easily available from these patients (than solid tumour samples), the behaviour of blood neutrophils may be completely different to that of TANs. When tumour samples are obtained, it must be noted that neutrophil function can be changed by the process of disaggregation used to extract the neutrophils. Finally, in human studies, a lot has been attributed to *ex vivo* T cell responses which may not reflect true physiology. For example, as previously described there are concerns about artefact that may be created by the methodologies used for *ex vivo* T cell proliferation studies.

Overall, there needs to be an unpicking of the various neutrophil subpopulations, and a better understanding of their evolving roles as cancers progress, at both primary and metastatic sites.

### Growing points: neutrophils as therapeutic targets and as possible playmakers in the biological response to both established and newer targeted cancer therapy

In terms of established therapies, neutrophils have been shown to be important players in the beneficial immune response to antibody-based cancer therapy,^48^ photodynamic cancer therapy^49^ and Bacillus Calmette–Guerin immunotherapy.^50^ However, a growing area of research is considering neutrophils as a therapeutic target themselves. Whilst the short-lived nature of neutrophils and their essential role in host defence against infection will need to be considered, nevertheless, targeted therapies relating to neutrophil recruitment, function and polarization (targeting the pathways mentioned in this review) may be an attractive add-on therapy to conventional treatments (i.e. chemotherapy/radiotherapy) and newer immunotherapies. For example, in terms of neutrophil recruitment, there is an interest in targeting the CXCR2 pathway.^51^ Relating to neutrophil polarization, there have been trials of TGF-β inhibitors as a cancer therapy.^52^ With regard to direct neutrophil–tumour interaction, there has been interest in the use of NE inhibitors.^53^

Finally, an expanding area of interest is understanding how neutrophils respond to newer targeted cancer therapies. Inhibitors of the receptor tyrosine kinase c-MET have been shown to inhibit neutrophil recruitment to tumours, with some concerned this may be detrimental,^15^ but others hopeful of benefit.^54^ As previously mentioned, neutrophils have been shown to express PD-L1, and therefore immune checkpoint inhibitors may have a role in reducing neutrophil suppression of T cell responses.

### Areas timely for developing research: metabolic programming of neutrophils in the tumour microenvironment

It has been shown that during tumour progression, TAN distribution is more within the tumour, and TANs develop pro-tumourigenic properties.^55^ It is known that the tumour microenvironment has altered oxygen and metabolite availability. Oxygen-sensing pathways and metabolic flux regulate neutrophil function and survival responses.^56,57^ There is literature regarding metabolic reprogramming of tumour-associated macrophages.^58^ Of note, MDSCs, which have infiltrated tumours, increase fatty acid uptake, and inhibition of fatty acid oxidation blocks their immunosuppressive function.^44^ Whilst there is extensive literature regarding the role of MDSCs in hypoxia, whether metabolite availability or hypoxia in the tumour microenvironment plays a role in the functional polarization of neutrophils is yet to be explored.

## Authors' biography

Robert Grecian holds the position of Edinburgh Clinical Academic Track Clinical Research Fellow and Respiratory Medicine Registrar. He is currently undertaking a PhD in Respiratory Medicine, exploring the role that neutrophils play in advanced non-small-cell lung cancer. His work is funded by Cancer Research UK.

Moira Whyte is the Head of the College of Medicine and Veterinary Medicine, and is the Sir John Crofton Professor of Respiratory Medicine, at the University of Edinburgh. Her research interests have focused on the molecular mechanisms of innate immune cell apoptosis in the context of chronic inflammatory lung disease.

Sarah Walmsley holds the position of Wellcome Senior Clinical Fellow, University of Edinburgh; Professor of Respiratory Medicine and Honorary Consultant in Respiratory Medicine. Her research focuses on defining how oxygen sensing and metabolic regulation influence key neutrophil functions and survival responses. She aims to validate the therapeutic potential of selective manipulation of these pathways, in patients with chronic inflammatory lung diseases.
